# Genome-wide integration site detection using Cas9 enriched amplification-free long-range sequencing

**DOI:** 10.1093/nar/gkaa1152

**Published:** 2020-12-08

**Authors:** Joost van Haasteren, Altar M Munis, Deborah R Gill, Stephen C Hyde

**Affiliations:** Gene Medicine Group, Nuffield Division of Clinical Laboratory Sciences, Radcliffe Department of Medicine, University of Oxford, Oxford, UK; Gene Medicine Group, Nuffield Division of Clinical Laboratory Sciences, Radcliffe Department of Medicine, University of Oxford, Oxford, UK; Gene Medicine Group, Nuffield Division of Clinical Laboratory Sciences, Radcliffe Department of Medicine, University of Oxford, Oxford, UK; Gene Medicine Group, Nuffield Division of Clinical Laboratory Sciences, Radcliffe Department of Medicine, University of Oxford, Oxford, UK

## Abstract

The gene and cell therapy fields are advancing rapidly, with a potential to treat and cure a wide range of diseases, and lentivirus-based gene transfer agents are the vector of choice for many investigators. Early cases of insertional mutagenesis caused by gammaretroviral vectors highlighted that integration site (IS) analysis was a major safety and quality control checkpoint for lentiviral applications. The methods established to detect lentiviral integrations using next-generation sequencing (NGS) are limited by short read length, inadvertent PCR bias, low yield, or lengthy protocols. Here, we describe a new method to sequence IS using Amplification-free Integration Site sequencing (AFIS-Seq). AFIS-Seq is based on amplification-free, Cas9-mediated enrichment of high-molecular-weight chromosomal DNA suitable for long-range Nanopore MinION sequencing. This accessible and low-cost approach generates long reads enabling IS mapping with high certainty within a single day. We demonstrate proof-of-concept by mapping IS of lentiviral vectors in a variety of cell models and report up to 1600-fold enrichment of the signal. This method can be further extended to sequencing of Cas9-mediated integration of genes and to in vivo analysis of IS. AFIS-Seq uses long-read sequencing to facilitate safety evaluation of preclinical lentiviral vector gene therapies by providing IS analysis with improved confidence.

## INTRODUCTION

Gene therapy is the introduction of exogenous nucleic acids into cells or organisms to achieve a therapeutic effect (1–3). A gene delivery vector is typically required to deliver the desired genetic material to cells, and viruses, based on their natural ability to enter mammalian cells, have been widely exploited for such purposes (4). Among the most popular gene delivery vehicles are genetically modified, replication-defective retroviruses. The expanding knowledge of virology and retroviral vectorology, and the increased understanding of genetic disease, resulted in the first clinical trials of gene transfer into humans (5). These early trials, however, raised issues regarding the safety of early vectors based on Moloney murine leukemia virus, due to vector toxicity and activation of proto-oncogenes caused by vector-mediated insertional mutagenesis (6). Despite these early set-backs, gene therapy has overall advanced considerably, spearheaded by research into vector biology in attempts to improve efficacy and safety profiles (7). The ability of lentiviruses (LV) to efficiently target and integrate into both dividing and non- or slowly dividing cells (e.g. stem cells and neurons), together with the development of self-inactivating (SIN) vectors, has aided the translation of LV-based therapies into the clinic (8). This includes multiple LV-based clinical trials targeting hematopoietic stem cells and T cells for a variety of diseases (9) and adoptive T cell therapy (e.g. CAR-T) as a promising oncotherapy approach.

Lentiviral vectors have been derived from not only Human Immunodeficiency Virus (HIV), but also from the Simian (SIV) and Feline (FIV) equivalents, as well as from Equine Infectious Anemia Virus (EIAV); such vectors are potent and versatile gene transfer tools used in multiple non-clinical and clinical studies (10–13). Previously, it has been demonstrated that different subtypes of retroviruses (e.g. alpharetroviruses, gammaretroviruses, lentiviruses) have distinct integration site preferences in humans and animals (14–16). Broadly divided into three categories, these viruses target either (a) sites within genes, (b) CpG islands and transcription start sites (17–19) or (c) display random patterns of integration with weak preferences for transcriptional elements. Integration of HIV-based vectors, led by host cell factor LEDGF/p75, are known to favor gene-dense regions distant from regulatory elements and sequences (20).

Since the advent of SIN vectors there have been no clinical trial reports of cases of insertional mutagenesis caused by LV integration, however, it is still of paramount importance to study vector integration sites to assess safety, prolonged genomic toxicity and posttranscriptional deregulation events (21–25). Several methods have been developed to investigate and report locations of newly integrated viral DNA. Most commonly, unidirectional linear amplification-mediated PCR (LAM-PCR) is combined with paired-end Illumina next-generation sequencing to quantify, analyze and map vector integration sites (26–29). Recently, another approach exploiting DNA-capture-Seq has been described (30).

Here, we report the development of a new method based on Nanopore MinION sequencing technology (31). While next-generation sequencing (NGS) methods rely on amplification and/or hybridization of regions of interest yielding short reads (in the range of 200–300 bp), for this work we exploited the ability of Nanopore to optimally sequence long DNA fragments. In this amplification-free method, genomic DNA (gDNA) samples are enriched for target sequences using SpCas9 ribonucleoproteins (RNP) targeting the integrated LV genome (32). Ligating adapter sequences to cut sites inside the integrated lentiviral genome generated by targeted Cas9 endonucleases enabled enrichment of regions of interest (i.e. lentiviral integration sites). The long read-length made possible by Nanopore sequencing enabled mapping of integration junctions with high confidence. All wet-lab steps in this protocol up to the final sequencing can be performed in a matter of few hours, using readily available and affordable reagents and sequencing equipment. Furthermore, data analysis can be performed on a standard personal desktop computer or laptop. As proof of concept we used gDNA obtained from cells infected with an HIV-based lentiviral vector pseudotyped with the widely-used vesicular stomatitis virus glycoprotein (rHIV.VSV-G), or with an SIV-based lentiviral vector pseudotyped with Sendai virus glycoproteins F and HN (rSIV.F/HN) which has been optimized for pulmonary gene transfer (13). Focusing mainly on human and murine lung cells and models, we carried out extensive integration site analyses and compared our method with a recent adaptation of the LAM-PCR protocol, shearing extension primer tag selection ligation-mediated PCR (S-EPTS/LM-PCR).

## MATERIALS AND METHODS

### Cells used

All cell lines were obtained from American Type Culture Collection (ATCC). HEK 293T (ATCC CRL-11268) cells were cultured in DMEM (Gibco) containing 2 mM l-glutamine (Gibco) supplemented with 50 U/ml penicillin, 50 mg/ml streptomycin (Gibco) and 10% heat-inactivated fetal calf serum (FCS). NCI-H441 (ATCC HTB-174) cells were cultured in RPMI-1640 supplemented with 2 mM l-glutamine, 10 mM HEPES, 1 mM sodium pyruvate, 4500 mg/l glucose, 1500 mg/l sodium bicarbonate, 50 U/ml penicillin, 50 mg/ml streptomycin and 10% FCS. Human surfactant air-liquid interface (hSALI) cultures were established by growing H441 cells in transwells in RPMI-1640 supplemented with 2 mM l-glutamine, 50 U/ml penicillin, 50 mg/ml streptomycin, 1% insulin-transferrin-selenium, 4% FCS, and 1 μM dexamethasone (Munis *et al.*, submitted). The hSALI cultures were maintained with an air/liquid interface for ≥2 weeks prior to LV transduction. LA-4 cells (ATCC CCL-196) were cultured in Ham's F-12K medium supplemented with 2 mM l-glutamine (Gibco), 50 U/ml penicillin, 50 mg/ml streptomycin and 15% FCS. All cell lines were cultured at 37°C in a humidified 5% CO_2_ environment.

### Virus production

Production of recombinant HIV vectors was performed using the four-plasmid transient transfection method previously described (33). Production of recombinant SIV vectors was performed using the five-plasmid transient transfection method (13). Briefly, LV-MAX™ Lentiviral Productions System was utilized per manufacturer's instructions. Virus supernatant was harvested 48 h post-transfection and purified via anion exchange chromatography and concentrated by tangential flow filtration. The vectors were formulated in TSSM (34), aliquoted and stored at –80°C. Functional titers were determined following transduction of LV-MAX™ cells with serial dilutions of the LV. Genomic DNA (gDNA) was extracted from cells 48 h post-transduction and purified using Qiagen DNeasy 96well Blood and Tissue kit and subsequently quantifying integrated viral and host cell genomes by qPCR using primers against WPRE (FW: TGGCGTGGTGTGCACTGT; RS: CCCGGAAAGGAGCTGACA; Probe: FAM-TTGCTGACGCAACCCCCACTGG-TAMRA) and hCFTR as endogenous control (FW: CTTCCCCCATCTTGGTTGTTC; RS: TGACAGTTGACAATGAAGATAAAGATGA; Probe: VIC-TGTCCCCATTCCAGCCATTTGTATCCT-TAMRA).

### Lentiviral transduction

Approximately 6 × 10^6^ cells were seeded into T175 cell culture flasks 24 h before transduction. Media was replaced with 25 ml of OptiMEM containing rHIV or rSIV-based LV at multiplicity of infection (MOI) ∼50. Following 6h exposure to the LV, OptiMEM was replaced with complete culture media. In contrast, hSALI cultures were challenged with LV at MOI ∼5 via administration to the apical side of the culture. After 6 h, the virus was removed. Approximately 72 h post-transduction, gDNA was extracted from cells using QIAamp DNA Mini Kit according to manufacturer's instructions.

### Calculation of vector copy number

Digital droplet PCR (ddPCR) was performed on a QX200 ddPCR System (Bio-Rad) according to the manufacturer's instructions to calculate VCN of transduced cells. All reactions on gDNA were performed with consumables purchased from Bio-Rad. The EGFP sequence was targeted to detect integrated LV copies (FW: CAACAGCCACAACGTCTATATCAT; RS: ATGTTGTGGCGGATCTTGAAG; Probe: HEX-ACAAGCAGAAGAACGGCATCAAGGT-Iowa Black FQ). Human CFTR (FW: CTTCCCCCATCTTGGTTGTTC; RS: TGACAGTTGACAATGAAGATAAAGATGA; Probe: FAM-TGTCCCCATTCCAGCCATTTGTATCCT-Iowa Black FQ) and mouse *Rpp30* (FW: CCAGCTCCGTTTGTGATAGT; RS: CAAGGCAGAGATGCCCATAA; Probe: FAM-CTGTGCACACATGCATTTGAGAGGT-Iowa Black FQ) genes were used as endogenous controls for human and mouse gDNA respectively.

### AFIS-Seq method

A TapeStation trace (Agilent Genomic DNA ScreenTape, Agilent, Waldbronn, Germany) showing the sizes of the DNA (comparable to an agarose gel) was performed to ensure that the extracted gDNA was of sufficient length to warrant progressing to the next step (suggested >50 kb to allow for DNA breakage during preparation steps prior to Nanopore sequencing leaving sufficient read length of cellular gDNA for efficient mapping, example TapeStation trace Supplementary Figure S1). The extracted HMW gDNA (10 μg) was end-protected by dephosphorylation with 15 units of Quick calf intestinal alkaline phosphatase (NEB, M0525S) in CutSmart buffer (NEB, B7204) for 20 min at 37°C and heat inactivated for 2 min at 80°C. Each of the four gRNAs (as annealed tracRNA and crRNA, both purchased from IDT, Leuven, Belgium) were complexed with a high-fidelity Cas9 mutant ((35) commercialized by IDT, 1081060) in an equimolar ratio to be delivered as a ribonucleoprotein complex. Sequences of gRNAs used: pGM285 (HIV.CMV.EGFP), Left 1: TTACCGTAAGTTATGTAACG, Left 2: AGATCCGTTCACTAATCGAA, Right 1: GCCCCGTTGACGCAAATGGG, Right 2: CGCGCCGAGGTGAAGTTCGA; pGM357 (SIV.hCEF.EGFP), Left 1: CGCTGCCGTCCTCGATGTTG, Left 2: CACGGGGCCGTCGCCGATGG, Right 1: GCTGTACAAGTAAGCGGCCG, Right 2: GCGCGATCACATGGTCCTGC.

A total of 6.2 μM of complexed RNP was used to cleave the four gRNA target sites per reaction. To the end-protected DNA, RNPs (1 μl of 10mM dATP) and Taq Polymerase (1 μl) were added, followed by gentle mixing by inversion, the tube was then placed in a thermocycler and incubated at 37°C for 60 min, to allow Cas9 to cut, and then at 72°C for 5 min, for efficient A-tailing by Taq polymerase. Adapters from the Oxford Nanopore Ligation Sequencing Kit (SQK-LSK109, Nanopore) were ligated onto the A-tailed ends using the manufacturer's instructions.

### Sequencing

Samples were sequenced using a R9.4.1 flow cell on a MinION Mk1B device following the manufacturer's protocol for ligation-based sequencing of gDNA in MinKNOW software version 19.12.2. Base-calling was performed with a CPU using Guppy version 3.1.5 for Windows with a Phred quality cut-off of >9. Prepared libraries were sequenced for ∼48–72 h.

### Analysis

To analyze the sequencing results, Phred quality score filtered sequencing reads were size-selected to ensure that the reads contained both the portion of the lentiviral sequence from gRNA cut site to the end of the lentiviral genome, and a portion of cellular genome (custom python script, Supplementary Figure S2), and then aligned to the proviral form of the lentiviral genome using Minimap2 (36), with the option to prevent multiple mapping events as the lentiviral sequence contains repeats (e.g. LTR). The sequences that aligned successfully to the lentiviral genome were then collectively mapped to the reference human (hg19), or mouse (mm9), genome using Minimap2. Commands used for read mapping can be found in Supplementary Figure S3.

### Read count determination for Ideograms

Read counts for a given locus were determined by counting the reads in a 50 000 bp window using a custom python script (Supplementary Figure S2). These values were used to generate ideogram figures in R using the RIdeogram package (37) (Supplementary Figure S4). Murine mm9 karyogram was generated in R using the commands found in Supplementary Figure S5.

### Calculation fold enrichment

The fold enrichment of lentiviral genomes sequenced through the AFIS-Seq method, over the predicted number of IS sequenced without enrichment given the observed sequencing depth, was calculated using the method below. The calculation is based on the number of sequence reads that successfully aligned to the lentiviral genome, divided by the expected number of lentiviral genomes sequenced with the total sequencing depth in each experiment. Number of sequencing reads was employed for this calculation rather than the number of base pairs of interest sequenced since the reads of interest contain a fixed amount of lentiviral genome sequence but vary in the length of cellular genomic sequence.

fold enrichment = (number lentiviral genomes sequenced)/(expected number of lentiviral genomes sequenced in total number of sequenced reads)

expected number of lentiviral genomes sequenced in total number of sequenced reads = $( {a*b} )\frac{{( {c*d} )}}{e}$

*a*: number of input genomes
*b*: vector copy number
*c*: total number of sequenced reads
*d*: average read size
*e*: total input bases based on input genomes

### Shearing extension primer tag selection ligation-mediated PCR (S-EPTS/LM-PCR)

Shearing Extension Primer Tag Selection Ligation-Mediated PCR (S-EPTS/LM-PCR) was performed by GeneWerk GmbH (Heidelberg, Germany) as described in Schmidt *et al.* (38) on gDNA extracted from HEK293T cells transduced with HIV or SIV lentivirus. Identical samples were split between AFIS-Seq and S-EPTS/LM-PCR experiments to allow for direct comparison. Primers complementary to the lentiviral LTR for the initial LM-PCR and two subsequent exponential PCRs were designed by GeneWerk GmbH. IS were identified by the closest gene name on the reference human genome (hg38) to the location where the vector genome was found.

## RESULTS

### AFIS-Seq efficiently detects integrated provirus DNA

**Figure 1.**
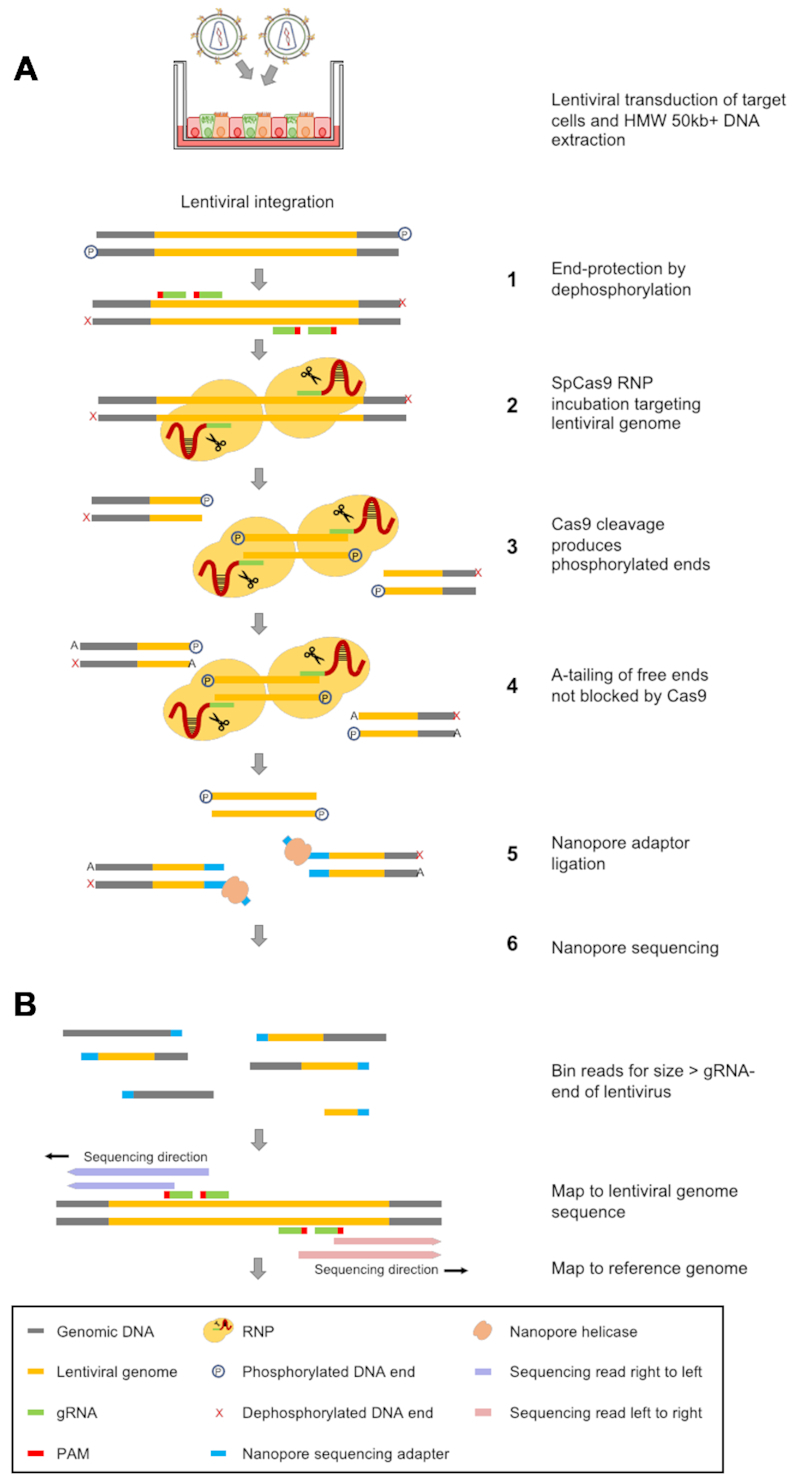
AFIS-Seq Pipeline. (**A**) Target cells are transduced with lentiviral vectors followed by high molecular weight (HMW) DNA extraction. Extracted gDNA is first end-protected by dephosphorylation (1) and then incubated with Cas9 RNP complexes designed to specifically cleave the lentiviral genome (2). Free DNA ends generated by Cas9 cleavage (3) are then A-tailed (4) followed by ligation of sequencing adapters (5). After several magnetic bead-mediated wash and elution steps, the DNA library is sequenced using a R9.4.1 flow cell on a MinION Mk1B sequencer. (**B**) A bioinformatics pipeline was generated for analysis of integration sites using custom python scripts, Minimap2 (36) and Samtools (55) software packages. Reads that are too small to include both the expected lentiviral sequence and sufficient genome sequence are removed in order to facilitate unambiguous mapping.

**Figure 2.**
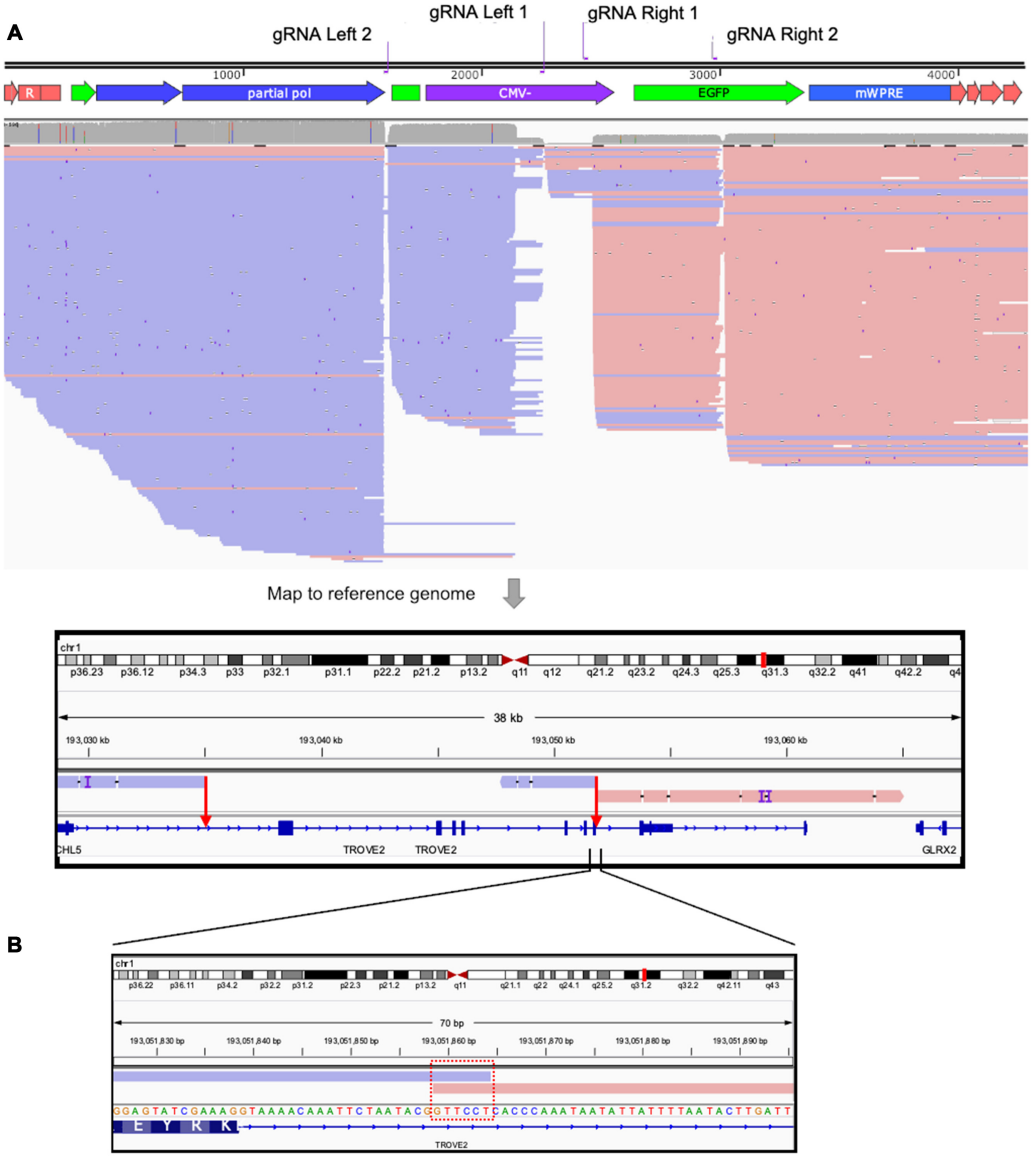
Example of AFIS-seq reads aligned to lentiviral genome and reference genome. (**A**) Reads that pass quality and size constriction (Figure 1B) are mapped to the integrated lentiviral genome sequence (modified from plasmid sequences to ensure SIN long terminal repeat (LTR) design). Reads that align successfully to the LV genome are mapped to relevant species genome (e.g. hg19 or mm9) for integration site identification (shown by red arrows) and analyses. (**B**) Highlights an integration event in which the lentiviral sequence is sequenced from both gRNA target sites outward into the cellular genome. The 6bp overlap seen here (highlighted in red box) is congruent with the 5–6bp duplication of cellular genomic sequence around the integration site that occurs when integration is mediated by lentiviral integrase (56).

In order to detect and analyze as many lentiviral integration sites as possible, we aimed to develop a method to enrich a DNA sample for our region of interest - the integrated lentiviral genome. The enrichment step begins with the incubation of end-protected gDNA with Cas9/guide RNP complexes (Figure 1A). For this, we designed a set of gRNAs targeting either the HIV or SIV vector genome (Figures 1A and 2A). Care was taken in the design of the gRNAs in order to minimize off-target cutting activity in the human and murine genome. Two gRNA target sites were chosen at either end of the provirus genome to provide Cas9-mediated cleavage redundancy. Guide pairs were designed to have both adequate spacing, to prevent potential steric hindrance, and to be a minimum of 500 bp removed from the integration junction to allow sufficient sequencing for efficient and confident mapping to the viral genome. After Cas9-mediated DNA cleavage, the free ends generated by targeted Cas9 endonuclease activity were then A-tailed to prepare for Nanopore sequencing adapter ligation. This method enables the selective ligation of sequencing adapters within the Cas9-cleaved integrated LV genome, to allow enrichment of the sample pool during sequencing after magnetic bead-mediated wash and elution steps. The gRNA targets on the provirus genome ends were placed on opposite strands relative to each other (Figure 1B) to increase sequencing reads in the direction of the cellular genome. As SpCas9 will remain bound to the cleaved DNA end located 5′ relative to the PAM site, A-tailing on that end will be reduced, thus achieving the observed directionality of sequencing from the provirus genome into the cellular genome (Figures 1B and 2A, red reads run from left to right and vice-versa for blue). Nanopore adapter containing DNA fragments are then sequenced in a R9.4.1 flow cell on a MinION Mk1B sequencer (Figure 1B).

Initial experiments were carried out on gDNA from HEK 293T cells transduced with either rHIV.VSV-G and rSIV.F/HN vectors. In our initial experiments using HEK 293T cells, libraries were prepared from 10 μg of total starting gDNA, resulting in 199 032 and 187 328 total reads for HIV and SIV vectors respectively. From these raw reads, we identified integration sites in both samples (Table 1). When corrected for integrated vector copy number (VCN), this yielded an approximate enrichment of 285-fold for HIV and 1612-fold for SIV. The average length of sequencing reads was ∼12 kb, which means that after subtraction of the sequence used to align to the lentiviral genome, an average of ∼11 kb of sequence was available for each IS to allow mapping to the cellular genome.

**Table 1.** Integration site enrichment identified using different vectors and cell models

Vector used	Cell line/model	Input DNA per reaction (μg)	Total number of reads^a^	Number of reads mapped onto integrated LV genome	Number of integrations identified	VCN/cell	Fold enrichment^c^
**HIV**	HEK 293T	10	199K^b^	939	692	20.55	285
**SIV**	HEK 293T	10	178K	3722	182	11.58	1612
**SIV**	H441	10	312K	2894	255	5.83	728
**SIV**	hSALI	10	602K	661	36	1.11	994
**SIV**	LA-4	10	285K	303	78	3.15	226

^a^Raw MinION sequencing reads.

^b^K: thousand.

^c^See Supplementary Table S1 for fold enrichment calculations.

VCN: vector copy number.

### AFIS-Seq can be used in various cell lines and models

The rSIV.F/HN vector is being developed for conducting airway disorders such as cystic fibrosis (13), and also a range of parenchymal interstitial lung diseases such as surfactant protein B deficiency. Therefore, to investigate the practical applications of the AFIS-Seq method, we expanded the target cell lines to include parenchymal lung cells, namely human NCI-H441 and murine LA-4 cell lines. In addition, to test AFIS-Seq in a physiologically relevant lung cell model we repeated the experiment using an air-liquid interface cell culture model we recently developed based on NCI-H441 cells. The human surfactant air-liquid interface (hSALI, Munis *et al.* submitted) recapitulates human lung parenchyma characteristics and constituent alveolar type I and II cells found therein. Utilizing all these models we could isolate rSIV.F/HN-mediated integration sites with up to ∼1600-fold enrichment of the DNA library from samples that were transduced with LV at multiplicity of infection (MOI) as low as ∼1 (Table 1).

### Integration site analyses indicate lack of preferential integration in different in vitro cell models

**Figure 3.**
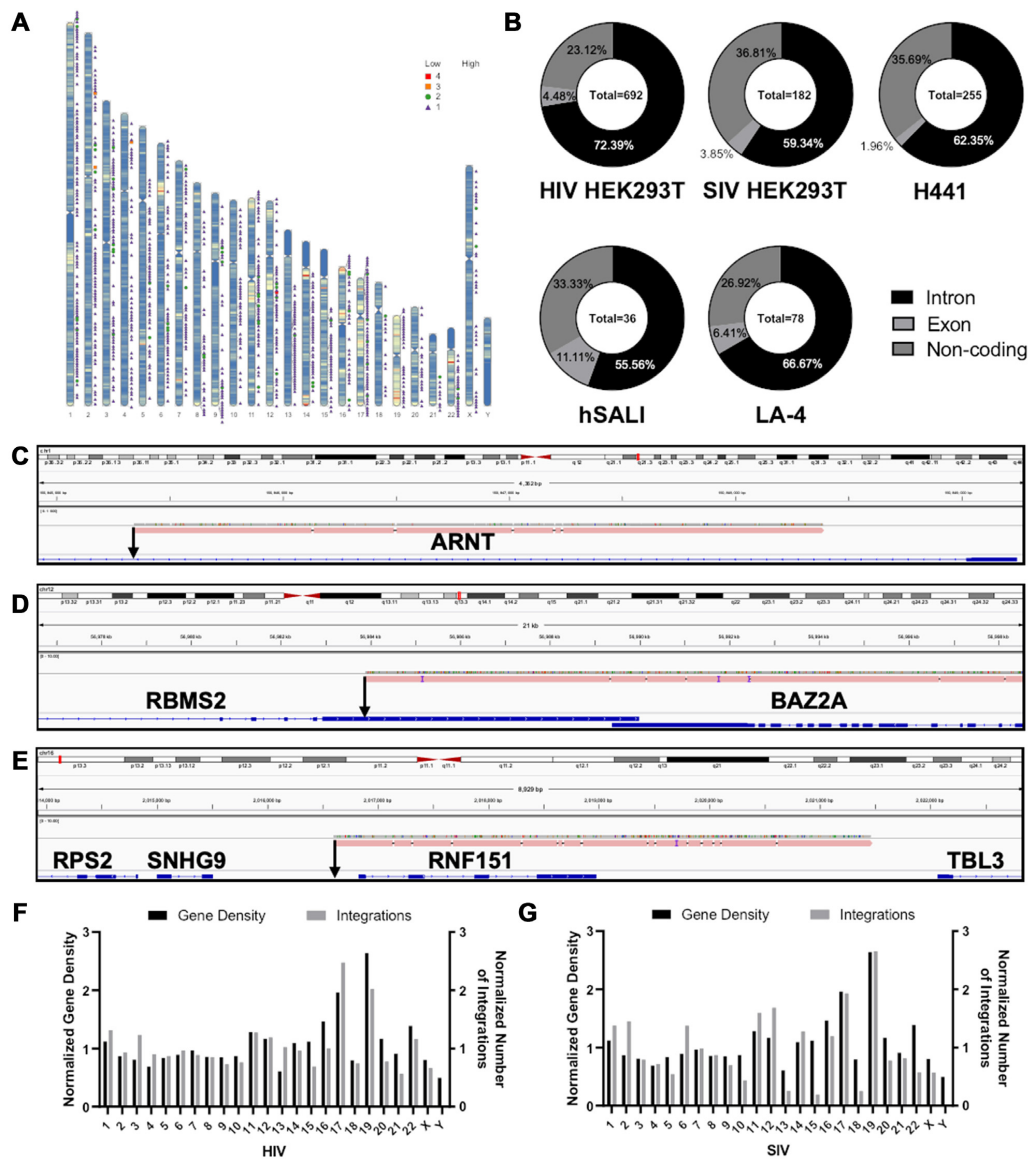
Positional Analysis of IS Identified. (**A**) Exemplar karyogram analysis of integration sites identified following lentiviral transduction. Identified integration sites plotted on karyogram of HEK 293T cells transduced with rHIV.VSV-G. Note the lack of integration sites in the Y chromosome as HEK 293T cells are of female origin (57). Ideogram is overlaid with the gene density of the respective species as a blue-red heatmap. Shape and color code indicating number of identified IS at a given location can be found at the top right corner of each ideogram. (**B**) Pie charts depicting locations of IS identified with respect to transcriptional units. Example reads indicating integration in (**C**) an intron, (**D**) an exon and (**E**) an intergenic region. Gene names are labelled in bold and IS are indicated by black arrows. Number of integrations identified in cells following transduction with (**F**) HIV and (**G**) SIV-based LV. The number of integration sites are normalized to the size of the chromosome in which they are found and graphed with gene density of each chromosome normalized to its size. Linear correlation analysis returned an R^2^ value of 0.64 and 0.61 for HIV and SIV respectively.

Further analyses were carried out on the identified integration sites. All samples were mapped onto either human or mouse karyograms to determine the overall distribution of the IS throughout the genome (Figure 3A and Supplementary Figure S6). Integrations were binned into 50 000 bp intervals by means of a custom python script (Supplementary Figure S2). Obtained values were used to generate ideogram figures using R software. Visual analysis highlighted a lack of conserved integration sites between cell models or vector types.

**Figure 4.**
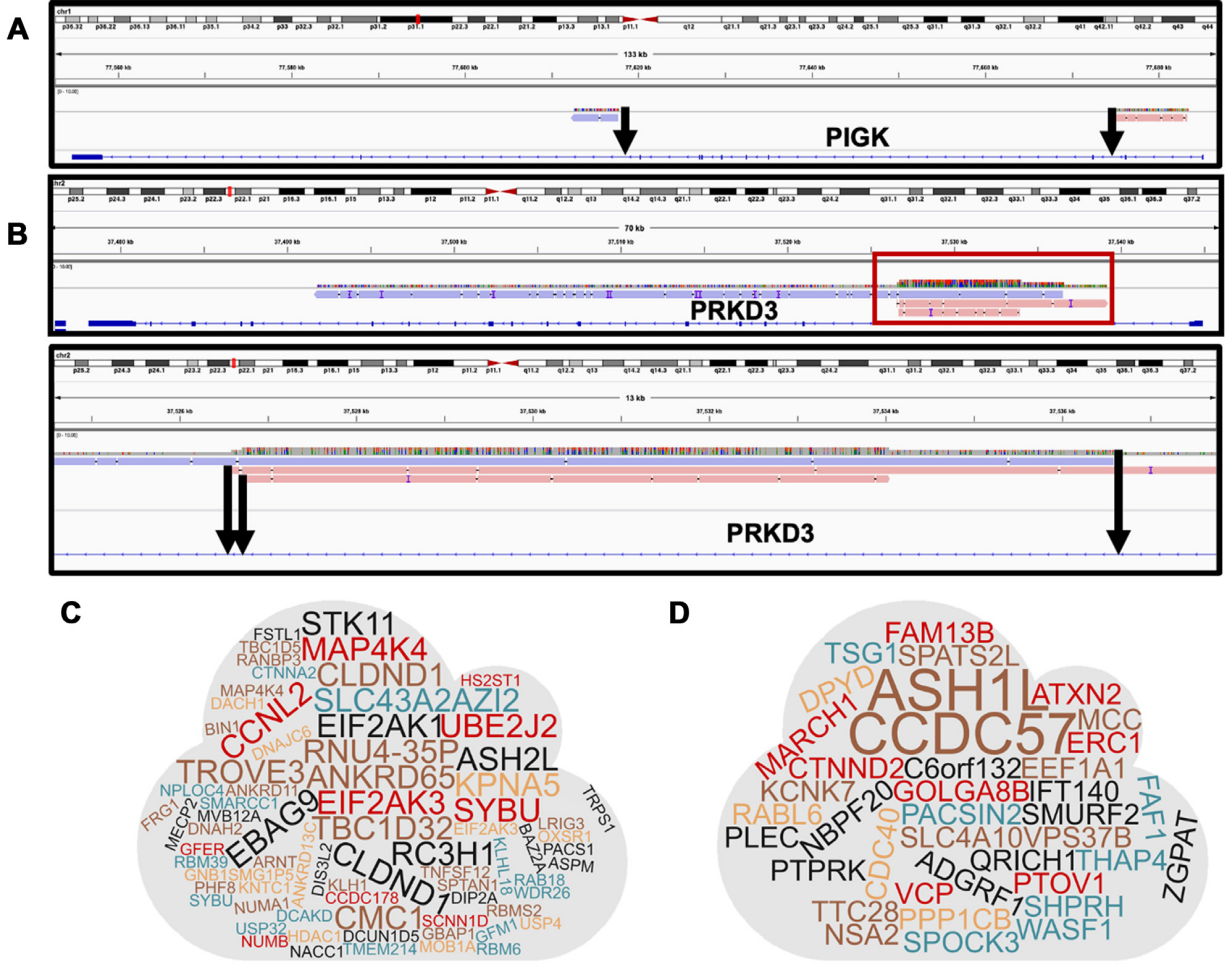
Identification of Multiple Integration Sites (MIS). (**A**) An example of a multiple integration observed where LV has targeted introns of the same gene body >60 kb apart. (**B**) An example of an integration site identified where multiple integrations were discovered in the same intron within 10 kb. Word cloud schematic of all MIS discovered in human cells following (**C**) HIV and (**D**) SIV-based lentiviral transduction. The sizes of the words within each cloud are proportional to the number of integrations found within the respective gene body.

Positional analysis of the IS revealed similar patterns in all five DNA libraries analyzed. On average, 73.4% of integrations were located in transcriptional units in line with the established lentiviral vector preference for integration around genes (Figure 3). Of these integrations near transcriptional units, 4.2% were in exons and 69.2% in introns. Approximately 26.6% of the integrations were found in non-coding (i.e. intergenic) sequences. Neither cell type nor species of origin appeared to influence these patterns. The chromosomal distribution of integration sites normalized to chromosome size likewise showed no clear indication of a skewed distribution for either HIV or SIV vectors (Figure 3B). Overall, and as expected, the number of lentiviral vector integrations in each chromosome correlated with its gene density (i.e. higher gene density resulted in more integrations) (Figure 3F, G). In addition, only a few multiple integration sites (MIS)—instances in which an integration occurred multiple times in the same gene body—were identified, confirming that integrations were overall random rather than targeted towards hot-spots. Two examples of MIS are shown in Figure 4A-B. As a result, no gene is overrepresented in our analysis of unique integration sites (Figure 4C, D). Furthermore, we did not find any strong evidence for mutations in the lentiviral genome over sequencing noise inherent to the sequencing method (data not shown).

### Comparison of genes targeted by different lentiviral vectors in different cell models

**Figure 5.**
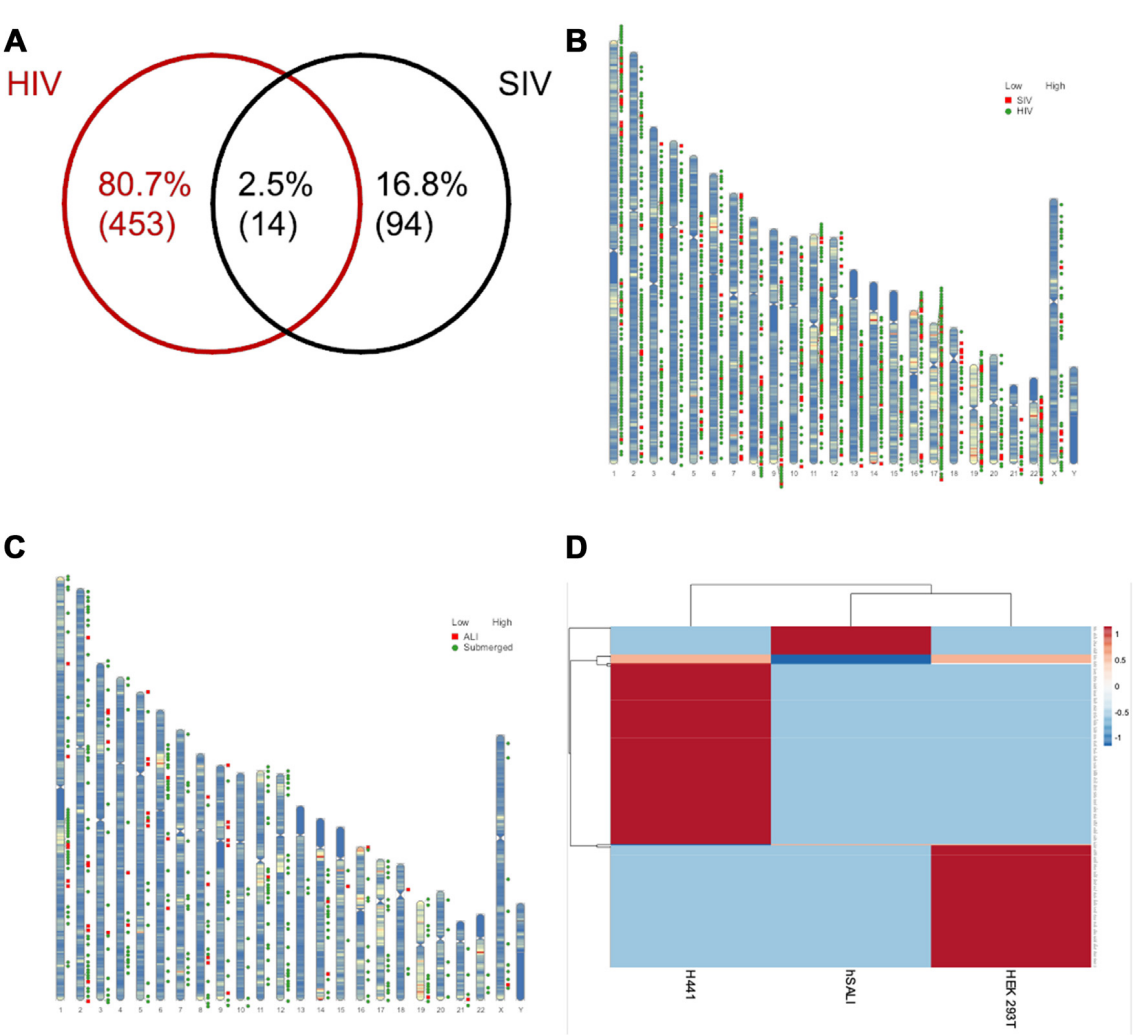
Comparison of Genes Targeted by HIV and SIV vectors. (**A**) Venn diagram depicting the shared integration sites (IS) (in gene bodies) identified in HEK 293T cells transduced with rHIV.VSV-G and rSIV.F/HN. (**B**) IS identified in HEK 293T cells transduced with HIV and SIV vectors superimposed on a human karyogram. (**C**) SIV vector IS identified in H441 cells cultured as hSALI and in submerged conditions superimposed on a human karyogram. (**D**) Heatmap analysis of all genes identified in the three human cell models following rSIV.F/HN transduction. Heatmap was made using ClustVis (58).

To address whether there is an overlap in the pattern of integration sites between lentiviral vectors from different species origin (i.e. HIV and SIV, originally isolated in humans and African green monkeys respectively) we compared the integration site distribution between HEK 293T cells transduced with rHIV.VSV-G and rSIV.F/HN (Figure 5A, B). As can be seen in Figure 5A, only 2.5% of integration sites occurred in the same gene.

Similarly, it was evident that there is no clear overlap in the genes in which rSIV integrates when used to transduce human lung parenchymal cells in either a submerged state (H441 cells) or grown as hSALI cultures and transduced from the apical side of the air-liquid interface (analogous to the lung lumen). The genetic makeup of these cells is identical, since hSALI cells are derived from H441 cells (Munis *et al.* submitted), implying that the cell type and the route of transduction do not influence the essentially random nature of lentiviral integration (Figure 5C). Further investigation of gene bodies in which integrations are found revealed that samples from hSALI cultures and H441 cells did not possess any common IS (data not shown). Furthermore, there was also minimal overlap with IS identified in HEK 293T cells transduced with rSIV.F/HN (Figure 5D). Together, these results confirm the intrinsic randomness of lentiviral integration highlighting the safety of this class of vectors. Although there is insufficient information to draw absolute conclusions, cross-correlation of identified gene-body targets with several oncological surveys (e.g Sanger COSMIC database) revealed no preference for integration near proto-oncogenes or tumor suppressor genes (data not shown).

### AFIS-Seq performs similar to S-EPTS/LM-PCR

In order to understand the strengths of AFIS-Seq method we performed side-by-side comparison analyses of HEK293T samples with shearing extension primer tag selection ligation-mediated PCR (S-EPTS/LM-PCR). S-EPTS/LM-PCR is regarded as superior to traditional linear amplification mediated PCR (LAM-PCR) as gDNA is prepared by random shearing through sonication, eliminating restriction enzyme biases (26,39,40). Potential IS are amplified twice in a nested PCR protocol and sequenced using Illumina MiSeq NGS technology. DNA preparation and sequencing were performed on each sample in triplicates (Table 2). From 272 965 and 188 477 sequencing reads a total of 7074 and 6018 unique IS sites were identified for HIV and SIV samples respectively. This translated to a successful IS mapping of 4% for HIV and 3% SIV samples using S-EPTS/LM-PCR while AFIS-Seq demonstrated slightly lower efficiencies of 0.4% and 2% for HIV and SIV samples respectively, likely attributable to lower enrichment efficiency in the amplification-free approach.

**Table 2.** Integration site detection efficiency of S-EPTS/LM-PCR

Sample	Input DNA per reaction (ng)	Total sequencing reads	Number of reads aligning to integrated proviral genome	% of input that is target (S-EPTS/ LM-PCR)
**HIV Repeat 1**	500	83 208	3645	4.381
**HIV Repeat 2**	500	80 346	3437	4.278
**HIV Repeat 3**	500	109 411	3842	3.512
**SIV Repeat 1**	500	72 114	2474	3.431
**SIV Repeat 2**	500	85 222	2348	2.755
**SIV Repeat 3**	500	31 141	1204	3.866

**Figure 6.**
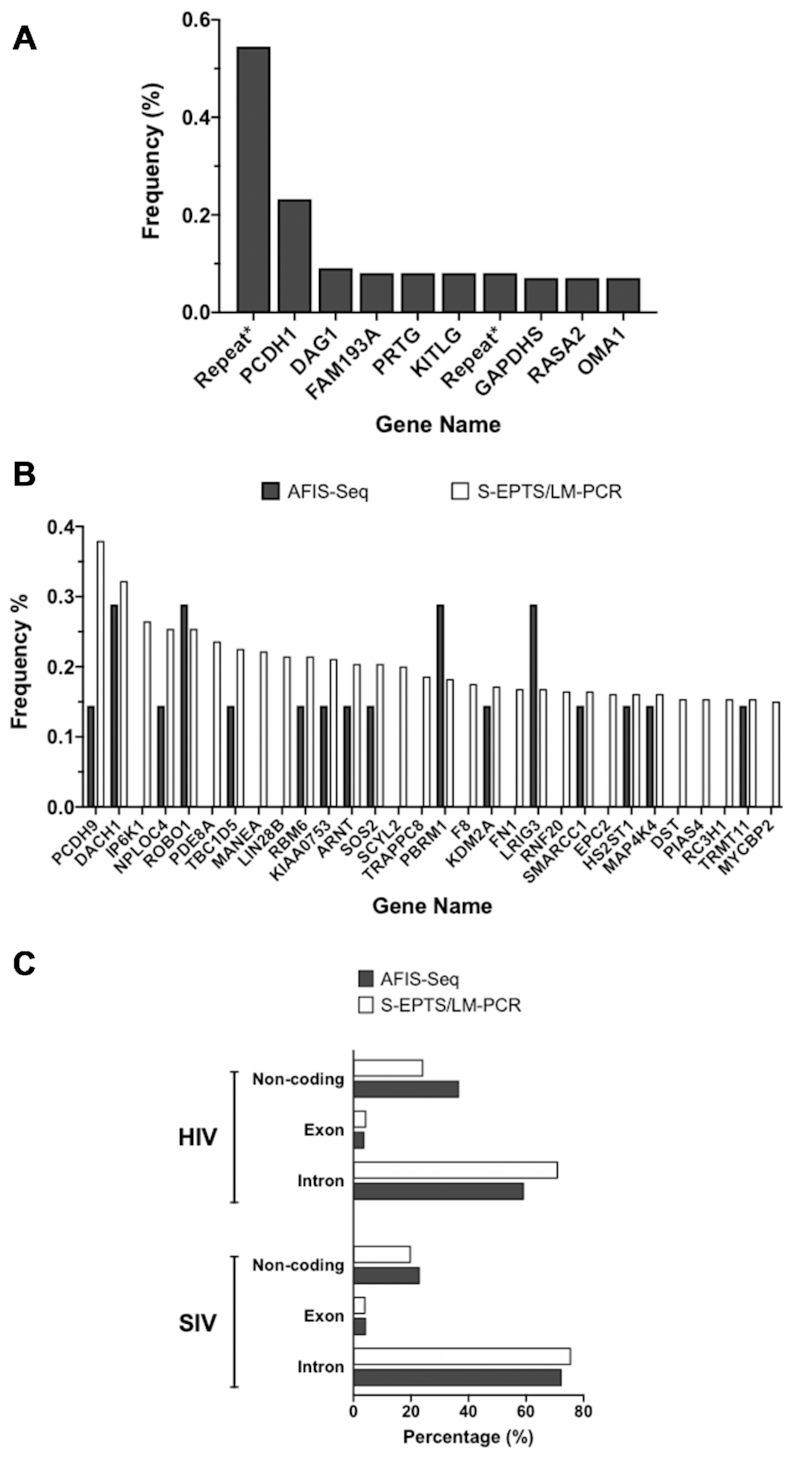
Comparison of IS profiles between S-EPTS/LM-PCR and AFIS-Seq in HEK293T samples. (**A**) Cumulative sequence counts and retrieval frequencies of the top ten most prominent IS detected in SIV HEK293T samples. The vector sequences that are not uniquely mappable to the reference human genome, denoted as repeats*, comprise the first and seventh most prominent IS hit in the sample. (**B**) Comparison of cumulative retrieval frequencies of 30 most prominent IS in HIV HEK293T samples. Data from individual S-EPTS/LM-PCR replicates were combined and plotted against the retrieval frequencies of the genes by a single AFIS-Seq run. (**C**) Comparison of integration sites with respect to transcription units between AFIS-seq and S-EPTS/LM-PCR on HIV and SIV samples.

However, during the S-EPTS/LM-PCR analysis, a high number of vector sequences were detected in both samples that could not be mapped to a unique location in the human genome. These sequences, potentially integration events in repetitive regions, accounted for the first and seventh highest frequency count in the SIV sample (Figure 6A, indicated as Repeats*). Furthermore, a high proportion of sequencing reads could be attributed to non-integrated lentiviral genomes (e.g. LTR circles (41,42)) (data not shown). Given the early harvest timepoint of gDNA samples (i.e. 72 h post-transduction) this was not unexpected and some LTR circles were observed in AFIS-Seq also (data not shown), however, these reads were easily eliminated in the AFIS-Seq approach during the bioinformatics pipeline by virtue of long read lengths associated with Nanopore sequencing. Furthermore, neither the AFIS-Seq nor S-EPTS/LM-PCR approach provided evidence of preferential integration in, or close to, proto-oncogenes and no single insertion site had a relative contribution of >0.3% of total IS. Comparison of the most prominent IS detected via S-EPTS/LM-PCR with AFIS-Seq data demonstrates that the majority of the top IS hits can be detected by both methods (Figure 6B and Supplementary Figure S8). Moreover, positional analysis of IS with respect to transcriptional units revealed highly similar patterns between AFIS-Seq and S-EPTS/LM-PCR (Figure 6C).

## DISCUSSION

The AFIS-Seq method described here is a sensitive strategy for the detection of lentiviral integration sites that does not rely on any form of DNA amplification. Instead, AFIS-Seq employs the targeted double strand break cutting action of SpCas9, to attach sequencing adapters to the provirus genome sequence of integrated lentiviral vectors allowing sequencing of the adjacent cellular genome by long-range Nanopore sequencing (Figure 1). Compared with the 3–7 days required for protocols based on LAM-PCR, the wet-lab portion of the AFIS-Seq protocol can be performed in only a few hours, using reagents and equipment readily available in a standard laboratory (i.e. not a core sequencing facility) and analysis can be performed on a basic Windows desktop or laptop computer.

Importantly, the lack of amplification steps in this enrichment protocol implies that this method should not suffer from stochastic amplification and/or GC-bias bias induced by PCR, which can be a significant issue with the LAM-PCR approach (43,44). On a similar note, sequencing adapters are ligated to the lentiviral genome end of the DNA molecule after Cas9 induces a DSB; this means there is no need for restriction enzyme digestion employed for adapter ligation as is customary in LAM-PCR protocols, thereby avoiding the bias that accompanies the requisite selection of restriction enzyme (45). The S-EPTS/LM-PCR used as a benchmark in this study does not employ restriction digestion but rather DNA fragmentation through sonication to create DNA ends for sequencing adapter ligation, overcoming this component of the procedural bias. However, IS recovery bias is not eliminated as S-EPTS/LM-PCR still relies on two successive rounds of PCR amplification for effective enrichment.

The AFIS-Seq method uniquely takes advantage of the long-read sequencing capabilities of the Oxford Nanopore sequencing platform. Protocols for detection of integration based on NGS are limited to 200–300 bp total read lengths, of which at least 100 bp is taken up by the lentiviral LTR sequence, not including the contribution of sequencing adapters and barcodes (27). This severely restricts the gDNA available to perform the genomic alignment. Using the method described here, an average of 11,000 bp are used for genomic mapping. While Nanopore sequencing is less accurate with respect to base calling than NGS alternatives, the increased read lengths achieved offset this concern and allow for high-confidence alignment even in regions of the genome where short reads often struggle, such as repetitive regions (46–48). Subsequently, S-EPTS/LM-PCR analysis was unable to map several IS for both HEK293T HIV and SIV samples, including the first and seventh most prominent hits in the SIV sample, due to this limitation (Figure 6A).

The experiments in this study were performed using an Oxford Nanopore MinION Mk1B device due to its affordability and accessibility. However, higher-throughput Nanopore platforms such as the GridION and PromethION are available which are capable of substantially greater sequencing yields (https://nanoporetech.com/products/comparison). When a second aliquot of gDNA from the same experiment (HEK 293T cells transduced with rHIV.VSV-G, Figure 3A) was sequenced on a second MinION flowcell, we identified a further 495 integrations, 92% of which (i.e. 437) were new, unique integration sites (data not shown), with only ∼8% of integration sites shared between sequencing runs, likely due to cell division after lentiviral integration. This implies that when sequencing capacity is ample, and sufficient DNA material is available, the number of unique integration sites detected by AFIS-Seq can be increased significantly. This observation also suggests that a single sequencing run on a MinION device does not capture the entire IS space, which could also explain the differences in IS profiles measured between cell lines in this study. However, the high VCN in our experiments could hamper the detection of the entire IS profile, as there are many more expected IS in the experimental samples than current sequencing methods can feasibly sequence. Importantly, little overlap in IS was found between replicates of the samples in S-EPTS/LM-PCR (66–98% unique IS between replicates), indicating that neither method is exhaustive.

The main limitation of the AFIS-Seq method is the requirement for a relatively large amount of genomic DNA. The described approach requires ∼10 μg of gDNA, whereas nrLAM-PCR or S-EPTS/LM-PCR only requires ∼500–1000 ng to detect a similar number of unique integration sites (27). The primary reason for the large input requirement is the absence of amplification. This implies that when fewer integration sites are present in the DNA sample, fewer will be sequenced. This is something that was observed in our experiments. Samples with lower VCN resulted in a lower number of unique IS (Supplementary Figure S5, *R*^2^ = 0.86). In line with this, when we performed AFIS-Seq on gDNA extracted from murine lungs transduced with rSIV.F/HN we were only able to identify <10 unique IS, likely due to the low VCN observed in the experimental samples evaluated (∼0.05, data not shown). This was expected, as rSIV.F/HN transduces only a percentage of the epithelial cells in murine lung (13), and the gDNA was prepared from bulk lung tissue without epithelial enrichment. This issue could potentially be overcome by a pre-enrichment strategy, such as the use of FACS to enrich for cells displaying a gene product (e.g. CAR-T molecule) expressed from the integrated sequence; but of course, this would not be applicable in every setting.

Selection of optimal Cas9 cut sites in the integrated lentiviral DNA is of paramount importance. For instance, the rHIV genome used in these experiments contained four optimal cut sites ∼500–1000 bp distant from the viral LTRs (Figure 1). This resulted in a high percentage of reads aligned with the viral DNA to map onto the human genome. In contrast, due to sequence limitations, we were not able to identify suitable Cas9 cut sites near the 3′ LTR in the SIV genome, creating a 3 kb long left arm (Supplementary Figure S7). This, unfortunately, led to the loss of some shorter reads in analysis, thereby reducing the number of unique integration sites identified.

Here, the AFIS-Seq method is applied to the detection of randomly integrated lentiviral genomes, but it can also be extended to other applications. For instance, it can be used to detect on- and off-target integration events in genome editing experiments, or in transposase-mediated DNA integration (transposition) events. Moreover, gRNA target design can be optimized such that a large portion of the integrated sequence is present in the sequenced reads, providing crucial information on the genetic makeup following the genome editing event. Current approaches such as ‘in-and-out PCR’ and UDiTaS (49) can only provide information on the integration junction, while targeted locus amplification (50), an alternative approach, struggles to resolve regions that contain repeats (51). Furthermore, an added feature of the AFIS-Seq approach is that post-hoc analysis of the raw Nanopore sequencing signal can reveal the presence of lentiviral genome DNA modifications (52–54), including CpG methylation of (for example) promoter sequences used to express a therapeutic transgene.

In conclusion, here we describe a new method to identify lentiviral integration sites. In addition to offering several practical advantages over existing protocols, the AFIS-Seq method provides multifaceted information from a single run, including: IS with high-certainty, provirus sequence, clonal abundance, and the presence of DNA modifications. Obtained IS profiles compare well between our method and the current state of the art LAM-PCR derived method S-EPTS/LM-PCR. We propose that AFIS-Seq, with the increased depth of sequencing it facilitates, will be extremely useful in analyzing heterogeneous samples obtained from preclinical and clinical specimens.

## DATA AVAILABILITY

All Nanopore sequencing data (Fastq files) are available on NCBI with BioProject ID PRJNA622800.

## Supplementary Material

gkaa1152_Supplemental_File

## Notes

Present address: Joost van Haasteren, California Institute for Quantitative Biosciences (QB3), University of California, Berkeley, CA, USA.
