# Supplementary material for: Genome-wide integration site detection using Cas9 enriched amplification-free long-range sequencing
Source: Nucleic Acids Res. 2020 Dec 8;49(3):e16. doi: 10.1093/nar/gkaa1152 (PMC7897500; doi:10.1093/nar/gkaa1152)
Supplement: gkaa1152_Supplemental_File [file gkaa1152_supplemental_file.docx]

***SUPPLEMENTARY MATERIALS***

**Figure S1. Example of TapeStation trace**


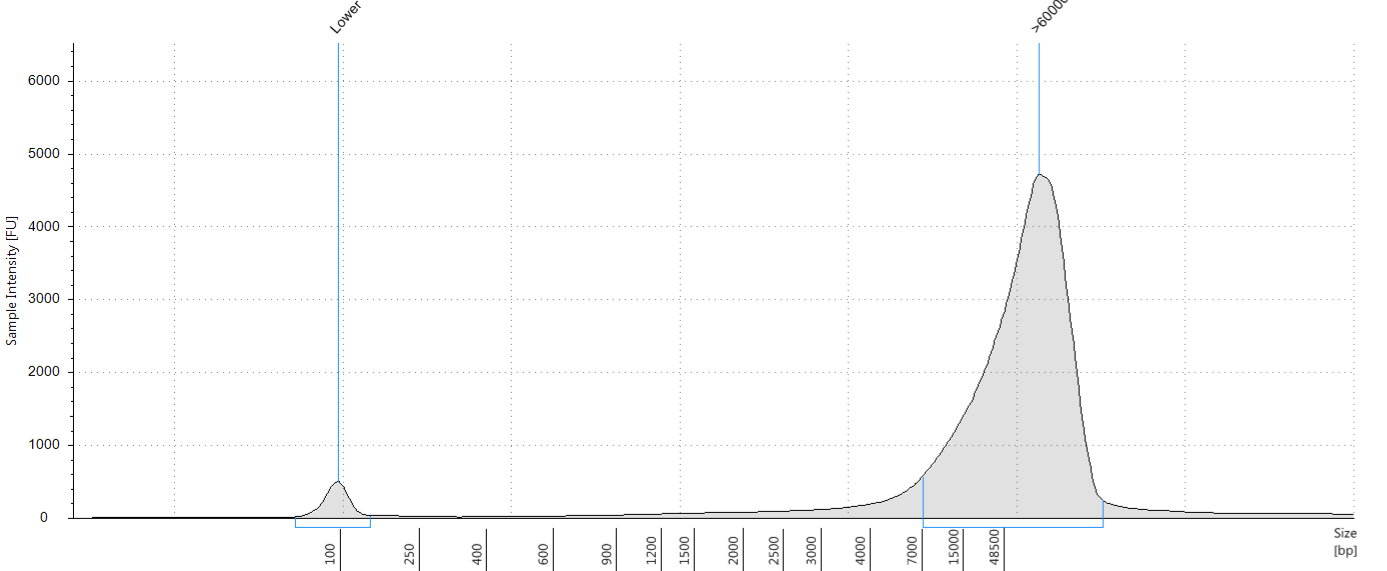


**Figure S2. Custom python scripts**

Size selection script (Python 3)

#!/usr/bin/env python3

import os

from Bio.SeqIO.QualityIO import FastqGeneralIterator

from Bio.SeqIO.FastaIO import SimpleFastaParser

from Bio.Seq import Seq

from Bio.Alphabet import generic_dna

from Bio import SeqIO

import glob

count = 0

total_len = 0

f = open('OUTPUT_FASTA.fa', 'w+' )

file_array = sorted(glob.glob("INPUT_FAST_FILES_*.fastq"))

for fastq_file in file_array:

with open(fastq_file) as in_handle:

for title, seq, qual in FastqGeneralIterator(in_handle):

length = len(seq)

if length >500: ## Edit this number to the required read size cutoff

f.write(“>” + title + “\n” + seq + “\n” )

Binned read counter (Python 3)

#!/usr/bin/env python3

import pysam

samfile = pysam.AlignmentFile(“BAM_FILE_OF_READS.bam”, “rb”)

window = 50000

f = open('COUNT_OUTPUT.tsv', 'w+' )

for i in range(len(samfile.references)):

    refname = samfile.references[i]

    seqlen = samfile.lengths[i]

    for j in range(1, seqlen, window):

        stop = j+window-1 if j+window-1 < samfile.lengths[i] else samfile.lengths[i]

        region_set = set()

        for read in samfile.fetch(refname, j, stop):

            region_set.add(read.query_name)

        #print("{}\t{}\t{}\t{}".format(refname, j, stop, len(region_set)))

        if len(region_set) > 0:

         f.write("{}\t{}\t{}\t{}\n".format(refname, j, stop, len(region_set)))

**Figure S3. Read alignment (Bash)**

#align to insert sequence

minimap2 -ax map-ont -N 0 INSERT_SEQUENCE.fa READS_FROM_SEQUENCING.fa > OUTPUT_ALIGNMENT_TO_INSERT.sam

#extract sequences from sam file

samtools fasta OUTPUT_ALIGNMENT_TO_INSERT.sam -F 4 > OUTPUT_ALIGNMENT_TO_INSERT.fa

#index reference genome for mimimap2

minimap2 -d mm9.mmi mm9.fa

#map sequences to mouse genome

minimap2 -ax map-ont -N 0 mm9.mmi OUTPUT_ALIGNMENT_TO_INSERT.fa > OUTPUT_MAPPING_TO_mm9.sam

samtools view -S -b -o OUTPUT_MAPPING_TO_mm9.bam OUTPUT_MAPPING_TO_mm9.sam

samtools sort OUTPUT_MAPPING_TO_mm9.bam  -o OUTPUT_MAPPING_TO_mm9.sorted.bam

samtools index OUTPUT_MAPPING_TO_mm9.sorted.bam

**Figure S4. Ideogram creation (R)**

install.packages("RIdeogram")

require("RIdeogram")

data(human_karyotype, package="RIdeogram")

data(gene_density, package="RIdeogram")

#load data from “binned read counter”

binned_reads <- read.delim("binned_reads.tsv")

#check if reads contain the correct information in the manner indicated below

head(binned_reads)

  Type    Shape Chr  Start End  color

1    1 triangle   1 150001 200000 cd0000

2    1 triangle   1 250001 300000 cd0000

3    2   box   1 300001 350000 ff7f00

4    1 triangle   1 600001 650000 cd0000

5    2   box   1 700001 750000 ff7f00

6    3   circle   1 750001 800000 00ff7f

ideogram(karyotype = human_karyotype, overlaid = gene_density, label = binned_reads, label_type = "marker")

###Generation of mm9 karyogram in R

#import gene annotation information from gencode

gene_density_mouse <- GFFex(input = "gencode.vM9.annotation.gff3.gz", karyotype = "m9karyo_genedens.txt", feature = "gene", window = 1000000)v

write.table(gene_density_mouse, "mouse_dens.txt", sep="\t")

#edit table to remove chr from chr1, chr2 etc

#read new table into gene density variable

mouse_dens <- read.table("mouse_dens.txt", sep = "\t", header = T, stringsAsFactors = F)

ideogram(karyotype = mouse_karyogram, overlaid = mouse_dens)

**Figure S5. VCN vs Integration Site Graph.** The relationship between the number of integration sites found through AFIS-Seq analysis and the vector copy number (VCN) of the analyzed samples is depicted. Linear correlation analysis returned an R^2^ value of 0.86.

**Figure S6. Karyogram Analyses of Integration Sites Identified Following Lentiviral Transduction.** Identified integration sites plotted on karyograms of HEK 293T cells transduced with **(A)** rSIV.F/HN. Note the lack of integration sites in the Y chromosome as HEK 293T cells are of female origin (52). Integration sites identified from *in vitro* lung cells/models: **(B)** H441, **(C)** hSALI and **(D)** LA-4 following rSIV.F/HN transduction. While the H441 cells are of male origin, the lack of integrations detected in the Y chromosome is expected due its small size and low gene density, as well as the limited sampling of the gDNA library. Each ideogram is overlaid with the gene density of the respective species as a blue-red heatmap. Shape and color code indicating number of identified IS at a given location can be found at the top right corner of each ideogram.

**Table S1: Fold Enrichment Calculations**

| **Sample** | **Total sequencing reads** | **Number of reads aligning to provirus genome** | **% of input that is target** | **Number of input genomes in 10** µ**g** | **VCN** | **Expected number of provirus genomes in sample** | **Fraction of genomes sequenced** | **Number targets expected in genome with given number of sequenced reads** | **Fold enrichment** |
| --- | --- | --- | --- | --- | --- | --- | --- | --- | --- |
| **HEK 293T HIV** | 93283 | 939 | 1.007 | 1.40E+06 | 20.55 | 2.88E+07 | 1.14E-07 | 3.29 | 285.52 |
| **HEK 293T SIV** | 178533 | 3722 | 2.085 | 1.40E+06 | 11.58 | 1.62E+07 | 1.42E-07 | 2.31 | 1,612.67 |
| **H441 SIV** | 568628 | 661 | 0.116 | 1.40E+06 | 1.11 | 1.55E+06 | 5.84E-07 | 0.91 | 728.75 |
| **hSALI SIV** | 303462 | 2894 | 0.954 | 1.40E+06 | 5.83 | 8.16E+06 | 3.57E-07 | 2.91 | 994.14 |
| **LA-4 SIV** | 233531 | 303 | 0.130 | 1.60E+06 | 3.15 | 5.04E+06 | 2.65E-07 | 1.34 | 226.91 |


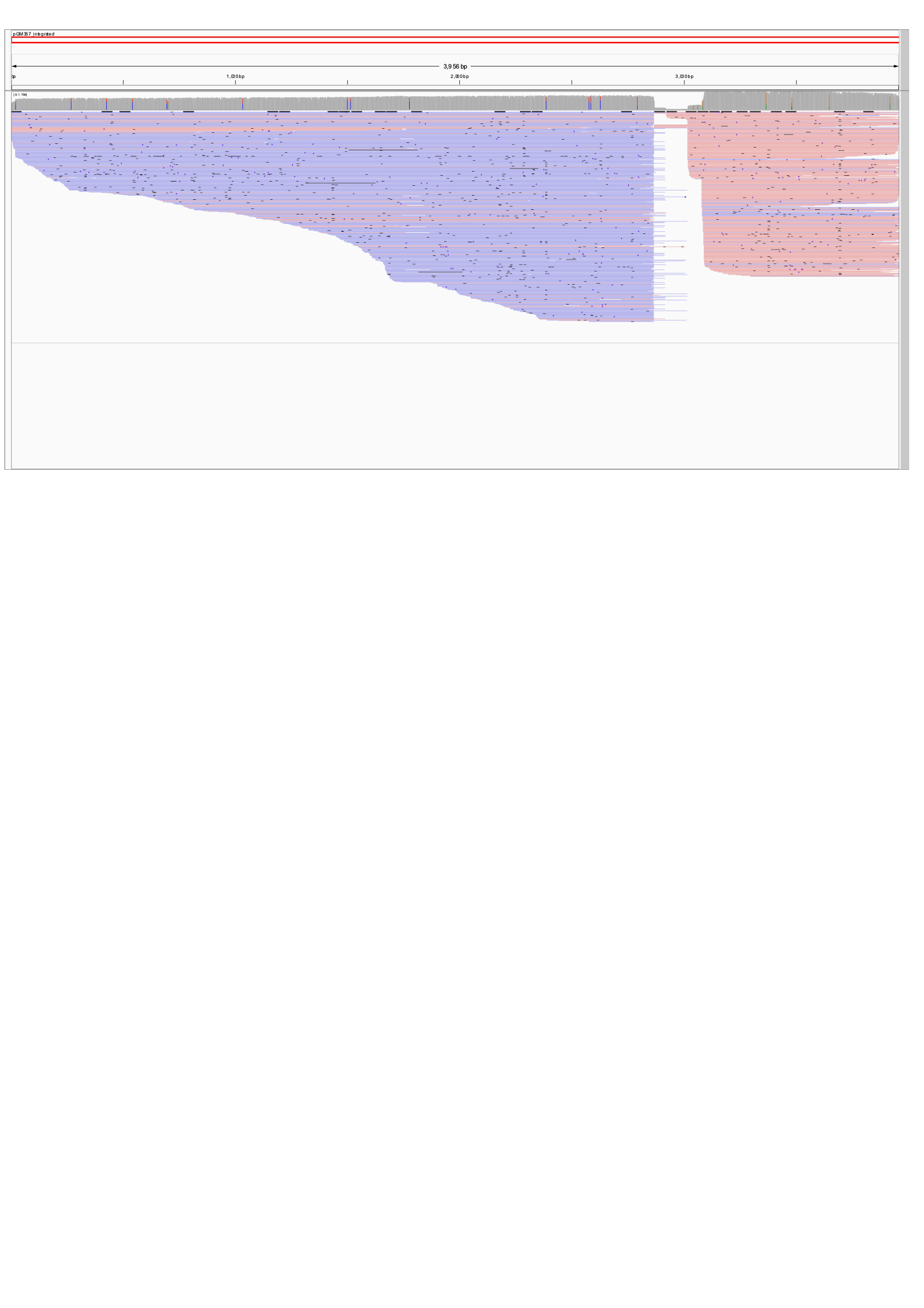

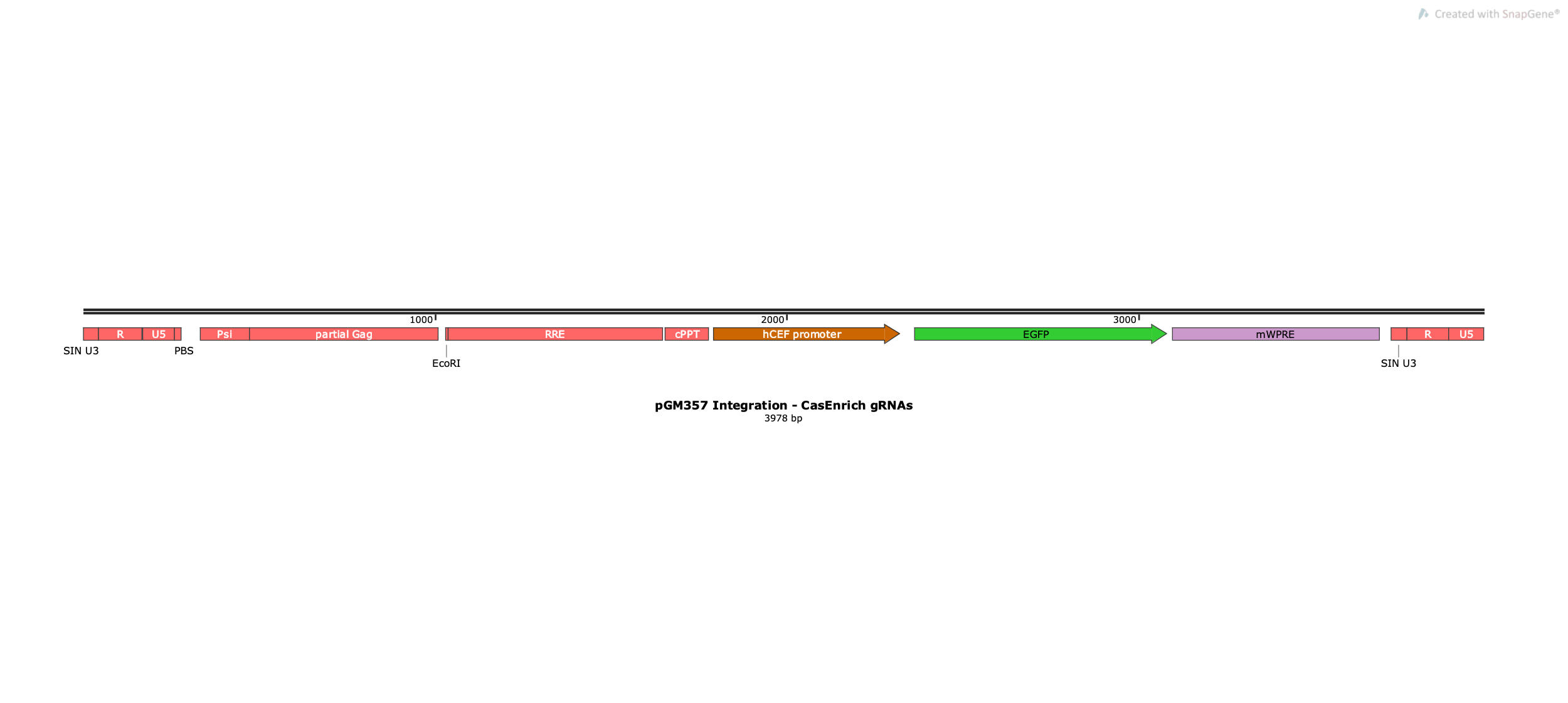


**Figure S7. Example Read Aligning to Integrated SIV Genome.** The top shows a schematic of the SIV provirus genome. Reads >500 bp are aligned using MiniMap2 are shown below. Blue reads align from right to left and red reads align from left to right. Note that the reads starting from the Cas9 cuts on the 3` end of the genome (red reads) end where the provirus genome ends and the chromosomal DNA starts whereas many of the reads on the 5` end (blue reads) end in the provirus genome because the spacing between Cas9 cuts and the end of the genome is too large.

**Figure S8. Comparison of Retrieval Frequencies of Thirty Most Prominent IS in SIV HEK 293T Samples.**
